# Designing and Evaluating a Prototype of a Trilingual Data Collection Tool for the Middle East and North Africa (MENA) Region to Collect Data About Violence Against Sex Workers: Multiple Methods Approach in User-Centered Design

**DOI:** 10.2196/65210

**Published:** 2025-08-22

**Authors:** Melissa Ditmore, Amal El Karouaoui, Jose Fernando Florez-Arango

**Affiliations:** 1 Clear-Eyed Consulting, P.O.Box 20853, New York, NY, 10009, United States, 1 3475609159; 2 Nawara Women's Network for the Middle East and North Africa, Casablanca, Morocco; 3 Department of Population Health Sciences, Weill Cornell Medicine, New York, NY, United States

**Keywords:** mobile health, sex work, user-centered design methods, usability, heuristic analysis, cognitive walkthrough, mHealth, data collection tool, violence, sex worker, stigmatization, health care workers, help-seeking behavior, human-centered design methods, heuristic evaluation, System Usability Scale, abusive, sexual violence, Middle East and North Africa, MENA

## Abstract

**Background:**

Sex workers face an epidemic of violence around the world. However, violence against sex workers (VASW) is underreported, and sex workers hesitate to report to the police because they are frequently punished; therefore, an alternative for reporting is needed. Sex workers also face stigmatization from health care workers, further discouraging help-seeking behavior or reporting in health care facilities. Sex workers have been recipients of services from nongovernmental organizations, typically related to HIV and sexual transmission of infections, but violence remains underaddressed.

**Objective:**

This study aims to apply human-centered design methods to adapt ReportVASW for use in the Middle East and North Africa and to evaluate the usability of the prototype interface and identify opportunities for improvement.

**Methods:**

Evaluation methods included cognitive walkthrough and System Usability Scale by 9 potential end users, and heuristic evaluation by 2 informatics professionals and 2 service providers.

**Results:**

This study explores ways to improve the trilingual prototype of ReportVASW, with particular attention to ways to improve the data collection tool. Multiple methods identified multiple issues to address. Heuristic analysis revealed 2 serious issues to address, with scores over 2.5 out of 4 in the tool used. The most serious problems identified in heuristic analysis were related to language, particularly the Arabic version. Translation issues were addressed before end user testing. End users were enthusiastic about the idea of a mobile tool to document VASW, provided it led to change. They gave ReportVASW a System Usability Scale of 91.4, above the 68 considered good. Even as end users were enthusiastic, they offered suggestions for improvement.

**Conclusions:**

Many opportunities to improve the interface were identified. Most changes are not overly complex, and the majority involve adapting the language used and improving the translation. Development of the trilingual ReportVASW data collection tool for the Middle East and North Africa region is worth pursuing.

## Introduction

Sex workers are frequently victimized by a variety of perpetrators [1] and 32% to 55% report at least one violent experience in the previous year [2], with numbers rising to near-universal in some locations [3]. Sex workers often do not report violent incidents [2,4]. Sex workers in Morocco “fear police too much to report violence.” [5] In fact, widespread criminalization in the Middle East and North Africa (MENA) region means that police may arrest them for sex work [5] instead of investigating violent crimes against sex workers; sex workers have been victimized or harassed by police [4-7]. Sex workers who seek care after victimization may not reveal their status as sex workers because health care providers may stigmatize and discriminate against them [8,9]. These factors culminate in an unfortunate lack of knowledge in the domain, including in the MENA region. Documenting violence against sex workers (VASWs) will help develop more effective responses to the health consequences of VASWs, including posttraumatic stress disorder [10] and HIV and other sexually transmitted infections [1,11]. Sex workers share information about perpetrators of violence among themselves [8], separate from reporting to law enforcement, in order to share information about violent experiences and to help others avoid their attackers.

While VASWs have been studied, few efforts to address this violence have been evaluated [12]. An exception is work by the Asia Pacific Network of Sex Workers, which commissioned an app called iMonitor+ that was designed for sex workers in Myanmar to help them report violence committed against them to an organization working with sex workers; the program was successful [13]. Other apps have been developed for sex workers in Cambodia [14] and South Africa [15]. No such app exists for use in the MENA region.

The lead author developed a prototype of a mobile health (mHealth) tool to collect data about incidents of VASW for use in the United States in line with the principles of user-centered design (UCD) for sex workers to report violence committed against them [16]. She then adapted this prototype for a trilingual data collection tool for the MENA region in partnership with Nawara Women’s Network for the MENA. Nawara engaged in a strategic planning process in 2022 [17] during which gender-based violence (GBV) was identified as an area of focus.

UCD was implemented in the adaptation for the MENA region. UCD with multiple methods, such as considering both end user and design use context, has been successfully used in mHealth [18,19], though it presents some challenges [20,21]. Formative research using UCD can be time-consuming, and users are not always easy to engage, but the literature shows the value of engaging end users in formative processes [20,21].

## Methods

### Overview

This section of the paper details the evaluation methods undertaken in usability testing with a multidisciplinary team of informatics professionals, Nawara Secretariat, and 10 potential end users based on the known theory that this number will generally expose the majority of problems with usability [22], in our attempt to evaluate the usability of the trilingual prototype of ReportVASW.

### Design Process and Prototype

#### Overview

The authors used UCD to design this prototype and its predecessor [16]. Ease of use is key because the user context is in the aftermath of a violent and possibly traumatic event. Ease of use and the lack of a learning curve are hallmarks of UCD. UCD is meant to improve usability by maximizing effectiveness, efficiency, and satisfaction of end users in the specific aim of the product in question. Early-stage implementation of UCD methods should reduce user error and limit the cost and time spent redesigning after developing software.

The specific principles of UCD include focusing on users and tasks, measuring usability empirically, and iterative testing of design and usability. UCD has been used with success to develop mHealth apps and health record systems [23,24]. These principles informed the specific methods used at each step. We followed the methodological steps of the UCD approach in our design process; these steps align with more specific methods: for example, contextualization using functional analysis and consulting potential end users; ideation through task analysis focused on the steps required for the end user to successfully complete the task; prototyping using representational analysis of the tool; and finally, usability testing using scenario-based testing and heuristic analysis.

#### Step 1: Contextualization

The authors seek to adapt a tool to respond to VASWs in MENA.

Contextualization included conducting a literature review about GBV in the MENA region, including the Nawara strategic plan [17]. The Steering Committee expressed interest in and support for adapting the existing data collection tool [16] for use in the MENA region.

A semistructured interview was conducted with 10 Nawara members to ask whether such a tool would be useful; members in Morocco and Sudan expressed great interest, and members from other countries were also interested, especially if the tool could be adapted for wider use after working with sex workers for GBV more generally.

#### Step 2: Ideation

We had an English-language prototype that was evaluated using task analysis and heuristic analysis [16,25]. Adaptation for the MENA region required assessing and refining the original language prototype, based on the increased cultural understanding gained from the literature review and consultation, followed by translation into the other 2 most common regional languages, French and Arabic. Members of the community from Sudan provided the initial Arabic translation, which was later reviewed by one member of the Nawara secretariat. French was initially undertaken by the lead author and revised by 2 members of the Nawara secretariat. The Nawara secretariat was particularly helpful because they communicate with members from across the entire Arab-speaking world, from as far east as the Persian Gulf and as far west as Mauritania and throughout the diaspora.

#### Step 3: Prototyping

The lead author developed the prototype in Qualtrics (Qualtrics International Inc; 29 New Mexico, 2009) web-based survey software, and created 3 versions—English, Arabic, and French—in March 2022, incorporating the Nawara logo in April 2022. Nawara members expressed a desire to have one prototype in all 3 languages together. The lead author then developed a fourth version, including all 3 languages, in April 2022.

#### Step 4: Representational Analysis

Representational analysis consisted of heuristic analysis undertaken by 3 authors and one colleague (2 informatics professionals and 2 colleagues from the Nawara Secretariat) in April 2023. The form used is a spreadsheet developed by the final author [26], adapted from another spreadsheet tool [27], in which a scale of 0‐4 is used to grade each issue, from minor (1) to catastrophic (4), and 0 is used to indicate disagreement that the point is an issue. The spreadsheet also contains a column labeled “proposed solution.”

The Nawara Secretariat members identified multiple language problems, especially in the Arabic version, and refined the language to be simpler for people with little education to understand and for Arabic to be understood across the region, followed by adjustments to the English version to remain in line with the French and Arabic versions. The prototype was updated to reflect these changes before the cognitive walkthrough and usability testing.

#### Step 5: Cognitive Walkthrough

Additionally, the Nawara Secretariat recruited a convenience sample of 9 end users who self-identify as female, both assigned female at birth and transgender women, with experience in a variety of sex work venues (internet escorts, brothel workers, open-area sex workers—cafes, streets, parks, etc) to test the usability by entering data from scenarios provided (scenario based testing) in a cognitive walkthrough [22], using the proposed application to enter data from a scenario taken from interviews with sex workers; all 10 agreed to do the walkthrough remotely. Multiple meetings with stakeholders were held in order to reach a broad range of stakeholders by gender, education level, type of sex work, and venue.

All end users recruited did the cognitive walkthrough in April 2024, in 3 sessions. Three scenarios used were taken from the lead author’s research [28-30], and 2 scenarios were written by Nawara based on MENA sex workers’ web-based experiences; all are in the Multimedia Appendix 1.

#### Step 6: Usability Testing

The end users recruited for the scenario-based testing then rated the data collection tool using the System Usability Scale (SUS). The SUS is a 10-item Likert scale, with each item’s score ranging from 0 to 4. The scale items were read aloud. Odd-numbered items are scored at the scale position minus 1, and even-numbered items are scored at 5 minus the scale position; the sum of the scores is then multiplied by 2.5 to obtain the overall score [31]. We searched the literature for an Arabic-language SUS but learned that translation had not been found to be acceptable despite translation of the SUS into other languages being acceptable and useful [32]. Considering this, Nawara translated the scale.

### Ethical Considerations

This protocol was submitted first to the CUNY School of Public Health and Health Policy Human Research Protection Program in December 2022 and to the Weill Cornell School of Medicine Institutional Review Board (24-07027685) in March 2023 and was judged exempt at both institutions. Nawara Women’s Network for the MENA recruited potential end users, specifically sex workers who identify as female, who were known to them and deemed unlikely to be adversely affected by the task of entering information about a scenario of VASWs. Informed consent was obtained verbally before beginning the cognitive walkthrough. Nawara was prepared to offer emotional support in the event of any adverse effects, and this was not necessary. Lunch and money for round-trip travel (averaging 100 Moroccan dirhams or approximately US $9) were provided for the 9 in-person testers. Generative artificial intelligence was not used in any portion of the manuscript writing.

## Results

This section of this paper conveys the results of the evaluation and will be organized following the same 6 steps used in the methods section.

### Step 1: Contextualization

The literature revealed that GBV is a problem throughout the MENA region at rates comparable to or higher than in other regions of the world [33,34], and that GBV and legal and social restrictions on women limit the economic development of countries in the MENA region [33]. Furthermore, economic constraints on women create vulnerability in other ways, including pushing women into informal sectors such as sex work. Finally, sex workers are singled out for interpersonal violence [5] by multiple actors, including law enforcement, family, and the public. All of these factors are compounded for sex workers [5,11], who typically come from the most vulnerable groups.

Nawara’s strategic plan [17] recommended addressing GBV for the most vulnerable women, including sex workers, migrants, and transgender women.

During the consultation, members of Nawara’s Steering Committee were enthusiastic about ReportVASW, and Nawara staff and leadership confirmed that information collected using ReportVASW could be used in multiple ways:

1. This site may facilitate information sharing in addition to documenting violence committed against sex workers and generate evidence to be used in reporting violent incidents in which sex workers are victimized.
2. Geolocation data about violent incidents can be used in the allocation of resources by organizations that work with sex workers, and in advocacy for additional resources.

### Step 2: Ideation

After Nawara Steering Committee members confirmed that ReportVASW could be useful for people in the MENA region, Nawara undertook Arabic and French translations and edits of the original English and French text, which were then used in the creation of 2 more prototypes, in Arabic and French. The wording of the introductory screen was modified both to be understood throughout the Arabic-speaking world and also to cater to regional sensibilities in phrasing.

Potential end users and cybersecurity experts agreed that using a website to collect data was more desirable than an app because it would be less likely to be noticed by police if their mobile phones were under surveillance.

### Step 3: Prototyping

**Figure 1.**
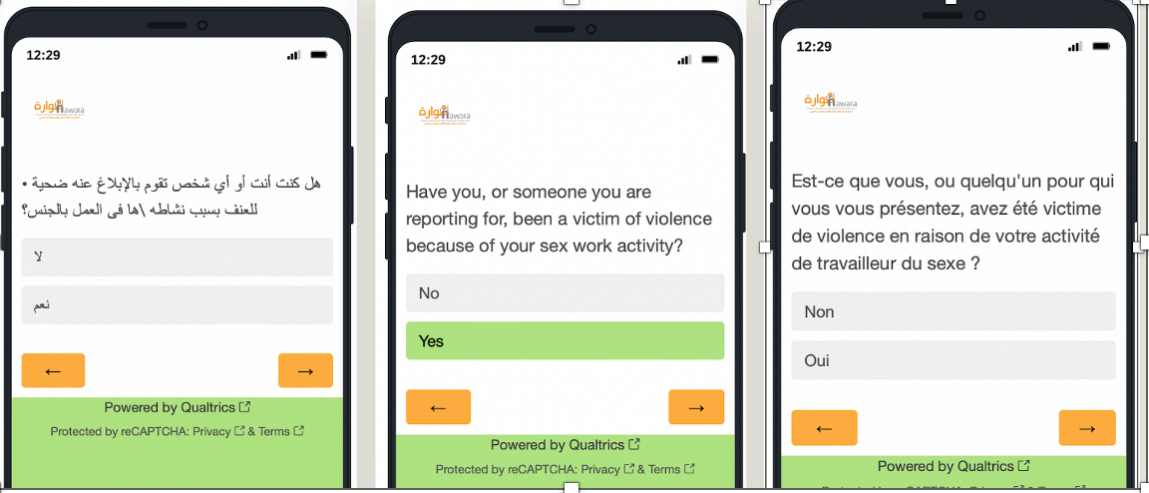
A prototype of ReportVASW presented separately in Arabic, English, and French.

The heuristic analysis and cognitive walkthrough using scenario-based testing methods generated similar assessments about the ease of use of the prototype and that the aesthetics were good; this overall agreement would seem to indicate that the findings reflect the actual usability of the prototype ReportVASW (Figure 1).

### Step 4: Representational Analysis

**Figure 2.**
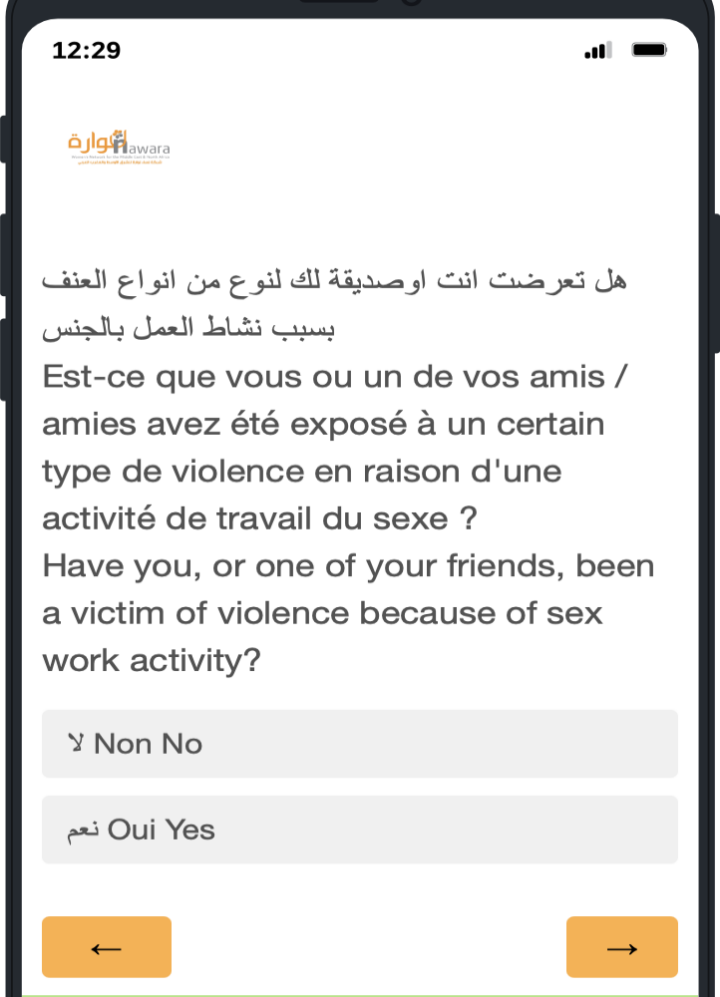
Final trilingual prototype of ReportVASW tested by end users (sex workers in Morocco).

Responses to the trilingual version (Figure 2) were compiled and average scores computed; heuristic analysis scores ranged from 0.5 to 3 and revealed that most problems were linguistic.

Two severe problems (with scores over 2.5) were identified. The first was to correct the name of the organization in Arabic on the first screen, violating the heuristic of visibility (average score 3). The second suggested problem and resolution was to add an Arabic label to the arrows because they are not the right direction for readers of Arabic (heuristic violation: Error prevention/Match/Language), with an average score of 2.5.

The number of violations of each heuristic listed in the Heuristic Analysis chart was counted. The most frequently violated heuristic was language (match the user’s reality) (n=8), stemming from multiple translations. Language was fine-tuned in Arabic, and then English and French were adjusted to match. These corrections were completed before end user testing (step 5) began.

### Step 5: Cognitive Walkthrough

Nine end users completed the evaluation, completing the task in under 2 minutes. End users confirmed the usefulness of an application like ReportVASW and said that it could be a viable tool for collecting information about violence committed against sex workers.

End users additionally confirmed that receiving a list of service providers and organizations offering services to sex workers would be helpful. This would need to be determined by location; Nawara has data for Morocco; this could be organized by city.

Some end users expressed a desire to share data about perpetrators, to prevent future exposure to violence, and one expressed a need to see the results of assistance, including help accessing services and possibly eventually seeking justice.

The cognitive walkthrough process delivered positive feedback, and users offered many ideas for improvements. Suggestions included the collection of additional information and comments about how they would benefit from this tool (eg, “If we had this five years ago, we could have prevented a lot of violence”), and what would be necessary to motivate them to use ReportVASW (eg, friendlier help from service providers in the aftermath of violent incidents).

End user testers of the prototype (Figure 1) recommended changes to data collection and follow-up as shown in Textbox 1.

Textbox 1. **Changes to data collection and follow-up.**

**Changes to data collection:**

- Add branching questions to follow negative responses to questions about reporting to the police to ask why people did not report to the police.
- Add branching questions to follow negative responses to questions about seeking health care to ask why people did not seek medical care, and include “stigma” among the choices.

**Changes to follow-up:**

- End users suggested following up to use the data collected for advocacy, including with police and health care personnel.
- End users saw the possibility of sharing information about violent incidents with sex workers in order to avoid perpetrators of violence.
- End users would like to see the ReportVASW function as a “warmline” with direct follow-up.

### Step 6: Usability Testing

ReportVASW scored 91.4 on the system usability; SUS scores over 68 are considered good [31] (Table 1). The tool scored neutral on consistency inspection, and all other responses were positive toward the application, with most being strong.

**Table 1.** System Usability Scale chart with number of end users (sex workers in Morocco) reporting each response.

	Strongly agree	Agree	Neutral	Disagree	Strongly disagree
I think that I would like to use this system frequently	7	2	0	0	0
I found the system unnecessarily complex	0	0	0	5	4
I thought the system was easy to use	4	5	0	0	0
I think that I would need the support of a technical person to be able to use this system	0	0	0	8	1
I found the various functions in this system were well integrated	0	9	0	0	0
I thought there was too much inconsistency in this system	0	0	0	0	9
I would imagine that most people would learn to use this system very quickly	6	2	1	0	0
I found the system very cumbersome to use	0	0	0	0	9
I felt very confident using the system	8	1	0	0	0
I needed to learn a lot of things before I could get going with this system	0	0	0	0	9

## Discussion

### Principal Findings

**Figure 3.**
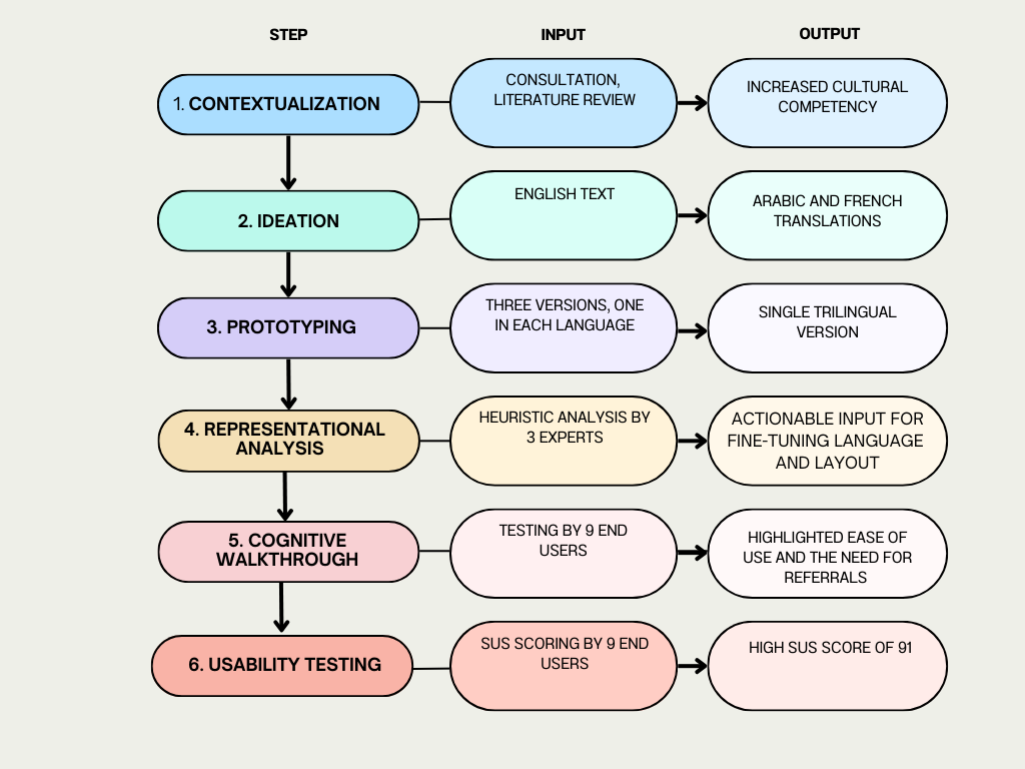
Flowchart of the process with input and output at each step. SUS: System Usability Scale.

Using multiple methods (Figure 3 contains a flowchart of the methods used) to evaluate the prototype enabled the collection of new information, including phrasing for screening questions, and positive reception of ReportVASW. New knowledge was gained from the evaluation, particularly through engagement with end users. We believe we have sufficient input and information to proceed to a pilot study.

Our experience reflects the literature [20,21] regarding the usefulness of multiple methods, despite challenges. Using multiple UCD methods at each stage of the process gave us richer information than we would have had with only one method at each stage, as others have described [18,19]. The time invested was significantly more than it would have taken with one method only, but the benefits are great because the information gathered will direct our next steps and reduce the chances of needing more changes later. The value of using multiple methods in UCD was clearly demonstrated in the process of designing and evaluating a prototype data collection to submit information about incidents of VASWs. Using multiple methods in the initial steps of contextualization and ideation led to multiple revisions in these early stages. Using multiple methods in prototyping and evaluating the prototype afforded the opportunity to collect informative input from people in different roles, including end users and informatics professionals. Results from each method aligned such that the representational analysis, consistency inspection, and heuristic analysis reinforced ways to improve the prototype, reinforcing the input from each source.

Partnering with Nawara enabled the adaptation and the discovery of the need for increased data literacy about digital personal security, including secure applications and browser history. This is in line with other literature that sex workers have adopted digital technology to address occupational safety and health [35] and shared information with each other [36] about benefits as well as new problems related to privacy [35,37]. We are unaware of other studies aside from our own implementing cognitive walkthrough with sex worker end users [16].

The iterative process of UCD contributed to the favorable evaluations of the trilingual version. UCD with multiple methods allows us to incorporate local sensitivity into the specific language and vocabulary used in this trilingual prototype, reflecting both success [18,23,24] and difficulties, including timing stakeholder involvement and managing heterogeneity among stakeholders [20,21] reported by others.

The heuristic analysis primarily identified problems with language, and end user comments offered confirmation of ways to make ReportVASW more helpful to end users; these solutions will be applied in the next steps. Additionally, UCD was useful in evaluating the ReportVASW prototype interface because adaptation is necessary to bring something initially designed for use elsewhere to the MENA context; input from end users will improve this adaptation. Evaluations of the interface using heuristic analysis and end user scenario-based testing will inform the revisions to the prototype.

The heuristic analysis offered actionable recommendations; most of the information offered was concrete and included suggestions that can be easily incorporated, for example, ways to formulate specific questions. Heuristic analysis further afforded interesting discussion related to the varied backgrounds of the analysts. For example, the most technically skilled of the analysts disagreed that some things merited high scores, because language issues are easy to address. However, other reviewers countered that the ease of addressing these language issues does not diminish the importance of making these changes for end users who read Arabic. Arabic reads in the opposite direction from English and French, and the directional navigation arrows point the wrong way in Arabic, such that clicking the arrow to advance for an Arabic reader actually goes to the previous screen. This may not be possible to address without a change in Qualtrics software to enable the addition of labels to the directional arrows in a variety of orthographies.

End user’s interested and substantive responses indicate a need for ReportVASW, provided that referral lists prove helpful and that the information collected is acted upon in advocacy. One complication is that few services for survivors of violence exist in Morocco, and therefore those available are overstretched.

Sharing the information collected about people who commit VASWs with people who may be targeted was identified as one possible benefit, with one end user saying, “If we had this five years ago, we could have prevented a lot of violence.”

Users said that ReportVASW is easy to use for those who are educated and literate, but that sex workers who are not able to read will have difficulty. It may be possible for others who know about their victimization to help them by reading the questions and entering the information for them, in line with sex workers’ peer-to-peer education promoting occupational health and safety [35-37]. In addition to issues of literacy, internet use is greater in wealthy countries than in low- and middle-income countries [38], although greater smartphone use everywhere may diminish this disparity; both text and voice messages have proven effective in reaching sex workers in low- and middle-income countries [39-41].

### Limitations

Double the ideal range of 4 to 5 end users [22] evaluated ReportVASW using cognitive walkthrough and SUS, leading to repeated input. We recognize the possibility of implicit bias in the implementation of the SUS scale, as the facilitator read the questions and asked for SUS scale responses orally, rather than end users writing in their responses.

### Conclusions and Next Steps

Follow-up is essential to the findings and implications of the project. We have received actionable recommendations through the cognitive walkthrough and the heuristic analysis that indicate clear, urgent next steps. The agreement between the task analysis of the cognitive walkthrough and the input from the cognitive walkthroughs and the heuristic analysis included many recommendations addressing aesthetics, usability, data collection, and other input about ways to improve uptake and also increase end users’ confidence that ReportVASW is benign and not used for surveillance. Immediate next steps based on this input include:

- Adding all the input in changes to screening and data collection offered by end users, including adding branching questions suggested during cognitive walkthroughs.
- Making the urgent changes identified in the heuristic analysis, including fine-tuning language.
- Developing ways to share information, including a “bad date report” and a resource guide to services.

The most important next step will be to link users to services that could be helpful in the aftermath of violence, including the long-term aftermath, involving long-term effects of violence such as chronic disease [1] and posttraumatic stress disorder [10]. Sex workers indicated that sharing links to services would meet this need for survivors who are literate. There are few services for sex workers, and many existing health service providers stigmatize sex workers, presenting an obstacle to access, and for this reason, end users must be involved in the decisions about services included in order to identify service providers that do not stigmatize or discriminate against sex workers.

These changes and next steps will be undertaken before piloting the data collection tool in Morocco. The pilot will collect data and provide an opportunity to ask more general users to rate ReportVASW using the SUS. Building more evidence will contribute to understanding the reasons for sex workers to report VASWs, so that the data collected can be used in advocacy.

We now have a product that we feel confident in testing. End users confirmed the need for an application like ReportVASW and that developing the data collection tool is worth pursuing, and informatics personnel reinforced the feasibility and offered insight to improve its design and utility. The use of multiple methods to evaluate the prototype contributed to a greater understanding than any single method alone.

## Supplementary material

10.2196/65210 Multimedia Appendix 1 Scenarios used for the cognitive walkthrough.

## Abbreviations

GBV: gender-based violence
MENA: Middle East and North Africa
mHealth: mobile health
SUS: System Usability Scale
UCD: user-centered design
VASW: violence against sex workers
