# Supplementary material for: Designing and Evaluating a Prototype of a Trilingual Data Collection Tool for the Middle East and North Africa (MENA) Region to Collect Data About Violence Against Sex Workers: Multiple Methods Approach in User-Centered Design
Source: JMIR Form Res. 2025 Aug 22;9:e65210. doi: 10.2196/65210 (PMC12373295; doi:10.2196/65210)
Supplement: Multimedia Appendix 1 [file formative-v9-e65210-s001.docx]

## Appendix. Scenarios used for the cognitive walkthrough

Scenario 1. I work in a brothel and we were robbed. One person came as a client, others followed and were let in by the first. They had guns and took our money. One person was hit, then everyone cooperated and they left. It took only a few minutes that went more slowly than any other time in my life. One of them had come as a [client] before and knew some of the layout.

Scenario 2. Someone pretending to be a [client] picked me up on the street, and drove me far away from everything. He beat me up. I was punched, pulled out of the car, kicked and pulled my hair. I have a black eye and a fat lip. I broke my shoe running away.

Scenario 3. This one cop, if he sees me, he keeps me in the squadcar. He has forced me to blow him, but not every time.

Scenario 4. My photos were used and shared on Instagram and they threatened to share the pictures with my family. I was blackmailed and had to have sex for free for them not to share the pictures with my family.

Scenario 5. I was in a café, using the wifi to set up an appointment with a client. Someone else on the her wifi tracked my conversation and threatened to share the conversation with my father. I paid money, I have no idea who did this.
